# DOX-DNA Interactions on the Nanoscale: In Situ Studies Using Tip-Enhanced Raman Scattering

**DOI:** 10.1021/acs.analchem.3c05372

**Published:** 2024-05-21

**Authors:** Katarzyna Majzner, Tanja Deckert-Gaudig, Malgorzata Baranska, Volker Deckert

**Affiliations:** † Department of Chemical Physics, Faculty of Chemistry, Jagiellonian University, Gronostajowa 2, 30-387 Krakow, Poland; ‡ Friedrich Schiller University Jena, Institute of Physical Chemistry and Abbe Center of Photonics, Helmholtzweg 4, Jena 07743, Germany; § Leibniz Insti-tute of Photonic Technology, Albert-Einstein-Str.9, Jena 07745, Germany; ∥ Jagiellonian Centre for Exper-Imental Therapeutics (JCET), Jagiellonian University, Bobrzynskiego 14, 30-348 Krakow, Poland

## Abstract

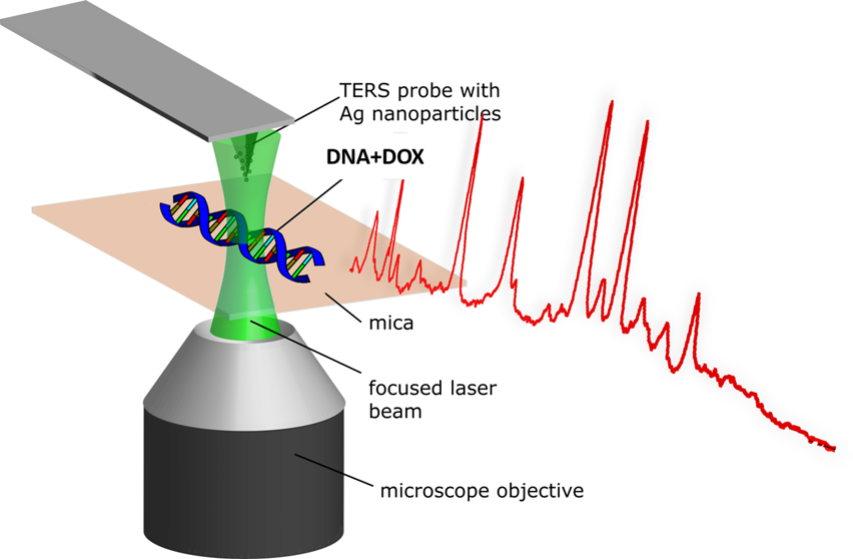

Chemotherapeutic anthracyclines, like doxorubicin (DOX), are drugs endowed with cytostatic activity and are widely used in antitumor therapy. Their molecular mechanism of action involves the formation of a stable anthracycline-DNA complex, which prevents cell division and results in cell death. It is known that elevated DOX concentrations induce DNA chain loops and overlaps. Here, for the first time, tip-enhanced Raman scattering was used to identify and localize intercalated DOX in isolated double-stranded calf thymus DNA, and the correlated near-field spectroscopic and morphologic experiments locate the DOX molecules in the DNA and provide further information regarding specific DOX-nucleobase interactions. Thus, the study provides a tool specifically for identifying intercalation markers and generally analyzing drug–DNA interactions. The structure of such complexes down to the molecular level provides mechanistic information about cytotoxicity and the development of potential anticancer drugs.

Chemotherapeutics, such as doxorubicin (DOX) or daunorubicin (DNR), are anthracycline antibiotics of high cytostatic activity and high efficiency. Their cytostatic function is related to forming a stable complex with the DNA helix, which prevents further division and leads to cell death. Such complexes are used in the treatment of various types of cancer, such as breast, pancreas, lung, malignant lymphoma, soft tissue sarcoma, and many others.^1,2^ Anthracyclines belong to the anticancer cycle-dependent and phase-specific drugs,^2^ which intercalate into the DNA helix. The resulting complex inhibits a variety of biochemical processes, such as DNA alkylation, DNA cross-linking, or the formation of free radicals damaging DNA.^2−5^ Anthracycline molecules are multifunctional derivatives of anthraquinone. DOX, the most common anticancer drug, contains a tetracycline ring structure with a daunosamine group attached by a glycosidic linkage. Despite a broad and relatively established treatment using DOX in antitumor therapy, its molecular mechanism of action is still not fully understood. This contribution used DOX as a model compound to study drug–DNA interactions *in situ* with tip-enhanced Raman scattering (TERS). In TERS, scanning probe microscopy is combined with Raman spectroscopy, simultaneously enabling topography imaging and spectra acquisition. The key part of TERS is a modified scanning probe, which was covered with silver nanoparticles in our atomic force microscopy (AFM)-based TERS setup. If such a metalized tip is irradiated with the appropriate laser wavelength, the electromagnetic field at the tip apex is amplified. Consequently, several orders of magnitude drastically enhance the band intensity of the Raman modes of molecules in the immediate vicinity of the tip apex.^6−10^ This phenomenon is based on the surface-enhanced Raman scattering (SERS) effect, where surface plasmons on rough metal nanoparticles are excited when irradiated with a laser wavelength close to their absorption maximum (plasmon resonance). Since in TERS (ideally), a single particle at the tip apex of an AFM or scanning electron microscope (STM) enhances the Raman signal, the spatial resolution is drastically increased.^8,11−15^ Thus, individual DNA or RNA strands can be probed with subnanometer precision.^11,14−16^

The present studies used TERS to identify and localize DOX molecules in DOX-treated calf thymus DNA. AFM data on DNA affected by DOX have already been reported, and Cassina et al. illustrated the consequences of drug binding on the morphology of a single DNA molecule (plasmid DNA, pUC19).^17^ Their work is based on topography changes and demonstrated that a high antibiotic concentration strongly affects the DNA conformation. In contrast, only a little effect was detected at low DOX concentrations (0.1 and 0.4 μM). With elevated DOX concentrations (0.7 μM), chain loops and overlaps of DNA become more likely, and the strands start to aggregate and entangle. A further increase in the concentrations of DOX (3.7 and 5.5 μM) results in aggregation of the DNA plasmid until the DNA collapses.^17^ Although the reported results provide helpful information on the effect of DOX on the DNA conformation, they lack chemical information.

In the first step of this work, SERS spectra of DOX were collected to define DOX Raman marker bands, enabling the differentiation from double-stranded DNA (dsDNA) signals in the subsequent TERS experiments on the DNA-DOX system. Our topography and molecular structure analysis results clearly show that the localization and identification of single intercalated DOX molecules in single double-stranded DNA was possible. The results are important for further pharmacological studies pursuing new therapeutic approaches involving drug–DNA interactions.

The following text is divided into two parts, both aiming at the molecular identification of DNA and DOX components. In the first part, SERS spectra of both DNA and DOX molecules are discussed to understand the plasmon-enhanced Raman spectra of both components. The second part focuses on the TERS spectra evaluation of single calf thymus DNA strands and DNA-DOX complexes. A sketch of the TERS experiment, the binding conditions in a DNA-DOX complex, and the AFM topography images of calf thymus DNA strands without and with intercalated DOX are shown in Figure 1.

**Figure 1:**
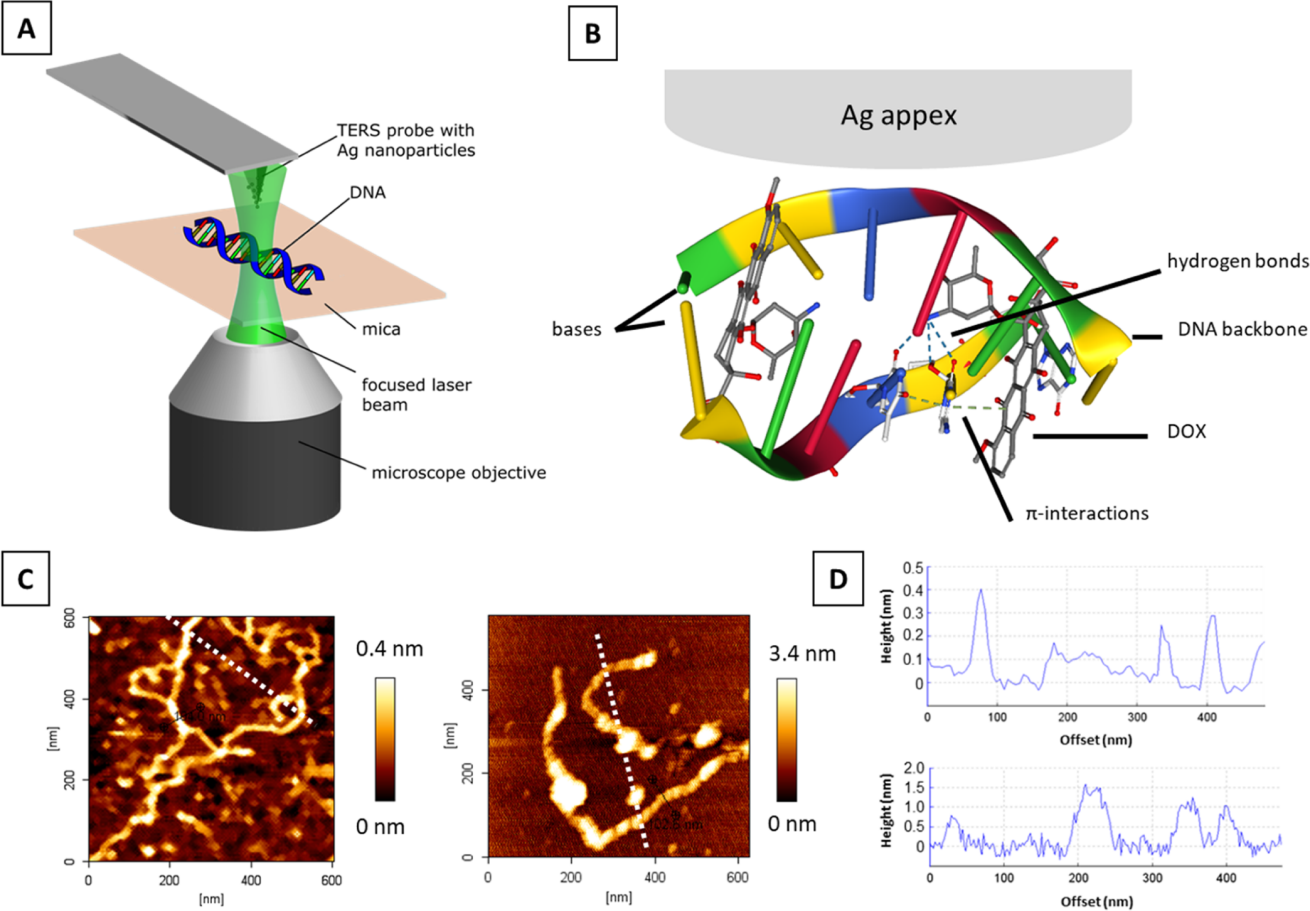
(A) Sketch of the experimental TERS setup. (B) Sketch of intercalated DOX in a DNA helix. The 3D DNA-DOX molecular structure was prepared in NGL Viewer4 based on ref (5) (Protein Data Bank ID: 151D). (C) AFM topography of control calf thymus DNA strands (left) and DNA incubated with DOX (right). The dotted lines indicate the height profile lines given in (D). (D) Profile lines across control calf thymus DNA strands (top) and DNA incubated with DOX (bottom).

The neat DNA sample was immobilized on freshly cleaved mica sheets (Figure 1a), and the topography was scanned in intermittent-contact mode to avoid sample damage (Figure 1c,d). The schematic DNA–DOX intercalation is sketched in Figure 1b. Due to the size of DOX molecules (1.5 nm), the strands were expected to expand at the corresponding sites, which was visible in the AFM topography as higher (brighter) spots. Figure 1C presents the AFM topography of calf thymus DNA strands scanned during the TERS experiment. The strand height was determined to ∼0.5 and ∼0.8–1 nm for DNA and DNA–DOX, respectively (see also Figure 1S in the Supporting Information) and agrees with the reported values for dsDNA.^17,18^ As expected, the height profile increased when DOX was intercalated (control DNA height ∼0.5–1 nm, DOX-treated height >1.2 nm). Changes in the morphology and the structural modification of the DNA strands are related to the intercalation of DOX molecules (slightly larger than single nucleotide), and they agree with previously reported values.^17,19^

The most popular spectroscopic methods for the studies on the formation of anthracycline-DNA adducts are resonance Raman, SERS, FTIR, CD, fluorescence, and molecular modelling.^20−21,22,23^ Despite their large information content, those techniques cannot localize single molecules in DNA strands due to spatial resolution limitations.

## Experimental Section

Supporting Information describes sample preparation protocols, AFM imaging procedures, TERS measurements procedures, and SERS measurements in detail.

## Results and Discussion

### SERS Characterization of DOX

**Figure 2:**
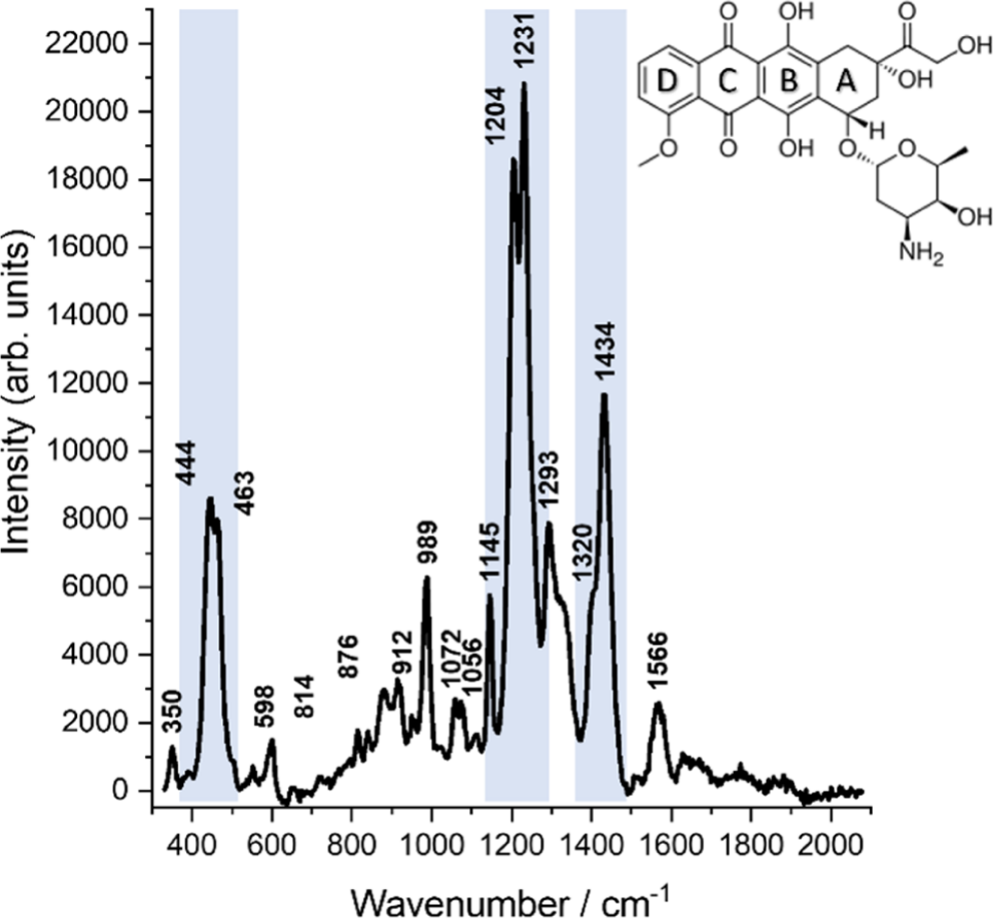
SERS spectrum of DOX adsorbed onto a silver island film. Characteristic Raman modes are highlighted. SERS spectra were recorded at λ = 532 nm (*P* = 750 μW, *t*_acq_ = 1 s).

Doxorubicin is a multifunctional derivative of anthraquinone composed of aglycone (4 rings connected to each other—A, B, C, D) and the sugar moiety daunosamine. The molecular structure of DOX is presented in Figure 2. The DOX molecule (Figure 2) contains four rings (chromophore) interacting with the DNA base pairs. Raman spectra of DOX recorded at 532 nm are dominated by fluorescence^20^ (an absorption maximum is located at 480 nm and assigned to a π–π* transition of the quinonoid structure^21^). This limitation was overcome in SERS, where fluorescence quenching was observed after the molecules were adsorbed on the silver nanoparticles (Figure 2, time-dependent SERS measurements of DOX are presented in Figure 2S in the Supporting Information). SERS spectra of DOX have already been published^22−25^ and reported.^26^ We referred to these data^20,21,23,24,26−30^ for the assignment of our SERS spectra in Table 1. The most prominent Raman bands are detected at ∼450 cm^–1^ (ring and carbonyl group bending vibrations^21,26^), 1204, 1231 cm^–1^ (hydrogen bond bending vibration (O–H···O); ring vibration; bend of C–O–H group^21,26^), 1434 cm^–1^ (aromatic ring vibrations^26^), and 1566 cm^–1^ (aromatic C=C stretch coupled with stretch vibrations of hydrogen-bonded carbonyl^21^). The most intense bands at 444, 1204, 1231, and 1434 cm^–1^ were reported as marker bands of DOX in biological studies.^20,33^ Additionally, these bands have already been proposed as an indicator for DOX in the DNA–DOX interactions.^21,34,35^

**Table 1:** Assignment of DOX Bands in the SERS Spectra (v, δ, ω—Stretching, Bending, Wagging Vibrations; a—Antraquinone Part, d—Daunosamine Part)^a^

(cm^–1^)	assignment of SERS bands^26,29^
350	δring, ωC–O–H (a), ωC–H2 (a)
**444**	**δ(C–O) ωO–H···O, ωC–H**
463	δring(phe), ωC–H (d), δC=O (a)
501	ωC–OH (a), ωC–O–C (a–d)
598	Skel. Def.
989	C–H2 (a), δC=O, δCOH (a)
1056	δ(C–H)
1072	ring breathing, δC–H (a), δC–C–C (a)
1145	δ(C–H)
**1204**	**δO–H···O, ring, δO–H (a)**
**1231**	**δO–H···O (a), δC–O–H (a), δC–H**
1293	δO–H**···**O, ring, δC–OH
**1434**	**ring–O, ring=O, δC–H2 (d)**
1566	ring breathing, ring(Phe)

a Dominant marker bands are highlighted in bold.

Our SERS spectra were used as a reference for the following TERS experiments. Since an overlap of DOX and DNA bands cannot be excluded, a combination of the dominating bands (bolded in Table 1) was used to evaluate the TERS data and unambiguously identify DOX.

### SERS Characterization of Calf Thymus DNA

**Figure 3:**
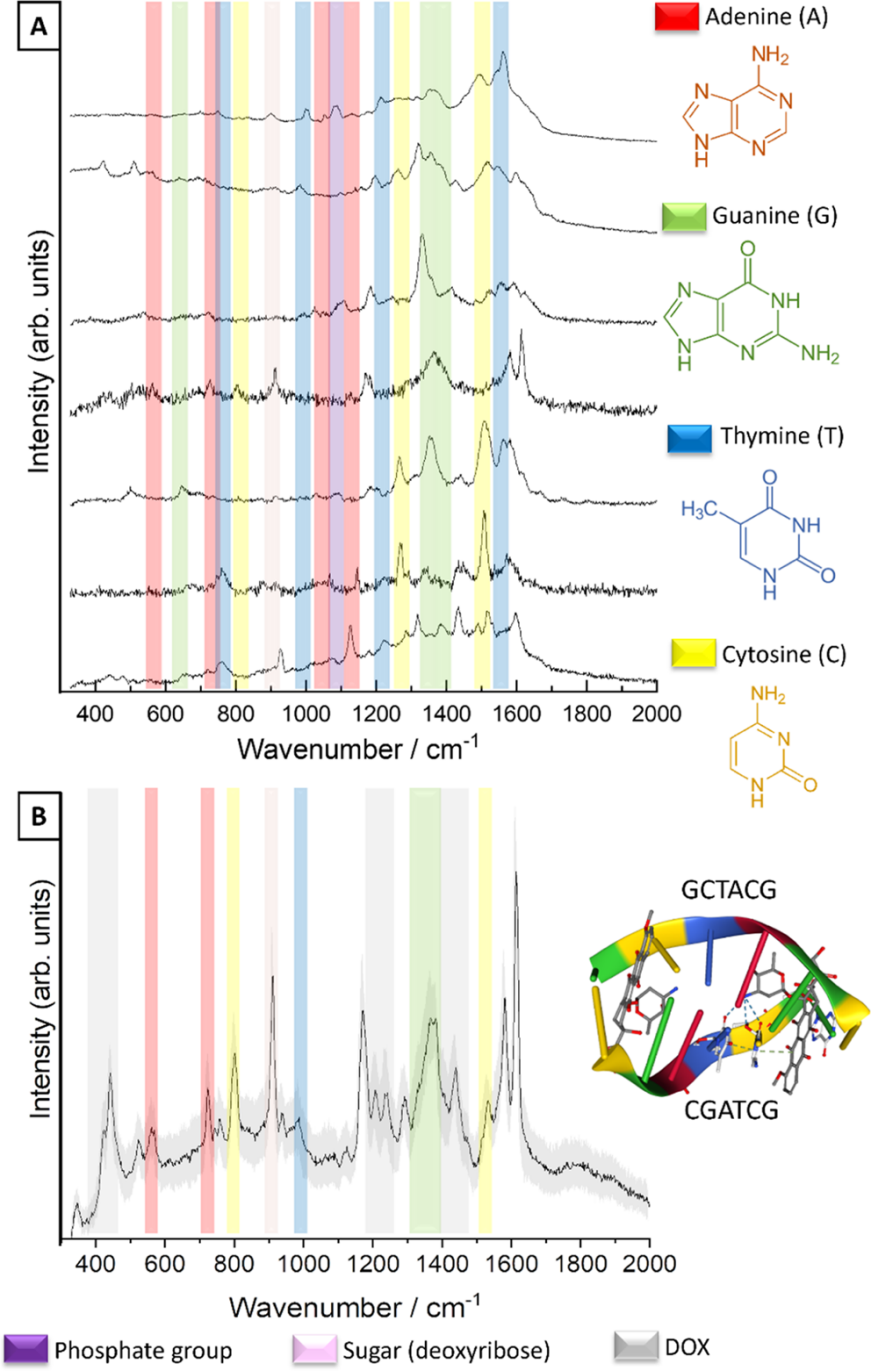
(A) Selected SERS spectra of calf thymus DNA adsorbed on a silver island film; (B) SERS spectrum of DNA with intercalated DOX (average of 20 SERS spectra; standard deviation presented in gray), characteristic Raman modes of DNA and DOX are labeled. SERS spectra were recorded at λ_exc_ = 532 nm (*P* = 750 μW, *t*_acq_ = 3 s).

For the experiments, double-stranded calf thymus DNA was chosen as a model since the mammalian DNA is analogous to human genomic DNA. DNA and nucleic bases have already been studied in numerous SERS works, and the band assignment has been confirmed.^36−42^Figure 3 shows typical SERS spectra from our experiments on untreated dsDNA samples. Since DNA molecules do not have a preferred binding site on the silver island substrate, the SERS spectra spectral fluctuations across the sample were observed. Nevertheless, the spectra allowed a clear assignment and distinction of nucleobases, deoxyribose, and phosphate moieties. The characteristic ring breathing modes of nucleobases were identified in all spectra (see Figure 3A) and enabled the reliable assignment of the respective bases (see Table 2). It is important to note that in the SERS and the following TERS spectra, the observed bands are a combination of vibrational modes rather than individual modes. In all nucleobases, the ring breathing modes can serve as marker bands and are assigned as follows: adenine: 720–730 cm^–1^; guanine: 650–675 cm^–1^; cytosine: 795–810 cm^–1^, and thymine: 740–760 cm^–1^.^43,44^

**Table 2:** Assignment of DNA Bands in SERS Spectra^a^

(cm^–1^)	assignment of DNA bands	ref
421	t (PO_2_)	(46)
492	G, T	(47,48)
506		
560	G (ring def); A ω(C–H, N–H)	(43,46)
645	G (ring breathing); A, C	(14,42,48)
720–730	A ν(ring)	(39,48,49)
760	ν(O–P–O), T (ring breathing)	(14,43,46)
785	ring breathing C	(37,47)
910	deoxyribose-phosphate, A/C/G ρ(NH_2_)	(46,51)
927	NH_2_ rk, sugar	(14)
983	T (out-of-plane ω (NH_2_))	(14)
996	C, T	(12)
999	deoxyribose	(48)
1024	deoxyribose-phosphate motion	(48,51)
1067	A ν(N-sugar)	(46)
1089	ν(O–P–O)	(48,51)
1127	A (C_8_−N_9_ str, N_9_–H, C_8_–H def)	(14)
1146	A(ν(C–N), δ(N–H, C–H)); deoxyribose ν(C–C)	(46,48)
1171	ν of sugar–phosphate backbone; A/G (C_5_–C_6_ str)	(14,49)
1184	δ(P–O–H), T	(51,52)
1197	C/T	(46)
1217/1221	T, ν (in-plane ring–CH_3_)	(14)
1268	C ν(ring), C/G νs (C–N)	(46,49)
1317/1320	A/G νs (C–N) (Im),	(46,48)
1332	purines (A and G) ν(C=N), ρ(CH) ribose, A ρ(C_8_H_8_)	(39,49,53)
1347/1363	G (ring mode), A ν(C–N, in-plane)	(46,53)
1368	A/T	(49,52)
1384	T(δ(NH), δ(CH_3_))	(46)
1434	δ(CH_2_) of deoxyribose	(48)
1448	ν(ring) of deoxyribose sugar	(49)
1498	G (C=N str (Im)); C	(14,48)
1507	C=C of ring A	(29)
1520	C (NH_2_ def), ν(CC)phenyl ring (out-of-phase), δ(CH_2_)crown (out-of-phase), ρ(CH) _Ph_	(48,53)
1552	T in-plane ν(ring)	(49)
1562	A/C/G/T (ν(ring _Py_)); ν(CC)_Ph_, A ν(C=N), A δ(NH)	(14,53)
1580	purines (A and G) ν(C–C)	(49)
1590	A/C/G δ(NH_2_), A δ(N_6_H_6_)	(49)
1614	C/G/T (ν_s_(C=O), ν(CC)), ν(CC)	(46)
1623	T ν(ring)	(48,49)

a Breathing—breathing mode of the 6-membered ring; ν—stretching; δ—scissoring; β—bending in-plane; γ—bending out-of-plane; ω—wagging; ρ—rocking; t—twisting; τ—torsion; Ph-phenyl.

As already mentioned, different orientations and/or packing densities of the DNA molecules on the SERS substrate affect the SERS spectra and are in agreement with earlier works.^38,39,44^ The present spectra show band position shifts and intensities depending on the coordination site and metal-sample interactions since DNA molecules usually bind to SERS substrates at an energetically favored geometry. It was proved that the position of the characteristic ring breathing mode allows for identifying binding nucleobases.

### Spectroscopic Characterization of DOX–DNA Interactions

As mentioned, the complexation between DOX and DNA is based on forming a complex between the DOX chromophore and distinct DNA base pairs.^2,54^ X-ray crystallography analysis demonstrated that the DOX–DNA complex is groove-reversal along with the DOX antenna (sugar moiety) in the major groove of the DNA strand. Notably, the antenna does not contact the DNA major groove, and the flanking base pairs sit in the minor groove.^55^ DOX is preferably located in areas with adjacent guanine-cytosine base pairs by forming hydrogen bonds with guanine.^56−58^ The SERS spectrum in Figure 3B of the DNA–DOX sample indicates the presence of DOX (see Table 1, Figure 2).

The comparison of the SERS spectra of free DOX and the DOX–DNA complex reveals a loss in band intensity (1206, 1240, and 1439 cm^–1^) in additionally to the lack of some DOX bands after complexation (i.e., 598, 989, and 1566 cm^–1^). It should be emphasized that the band intensities shown in Figure 3B at 1206, 1240, and 1439 cm^–1^ significantly decreased. Since those bands are related to the chromophore’s ring stretching vibrations, they can be regarded as markers for DOX–DNA interactions.

Surprisingly, spectral changes in Figure 3B mainly occurred in the band intensity but not the position. Since the chemical environment of DOX has changed upon complexation, some vibrational modes were expected to be affected, resulting in slightly changed band positions compared to the free molecule. Since this was not observed, the spectra were assumed to contain contributions from both complexed and free DOX. At this point, it has to be mentioned that free DOX was always present in the DOX–DNA solution. Regarding the intercalated DOX, it seems that rings inside the DNA double helix are no longer accessible for SERS enhancement. As a result, chromophore-related bands are detected with lower band intensity. Similar observations have been reported previously.^29^ Since SERS spectra always contain averaged information about all molecules in the laser spot, no differentiation of free DOX and DNA–DOX was possible. TERS experiments were performed to identify DOX in the DNA strand precisely.

### TERS Measurements

**Figure 4:**
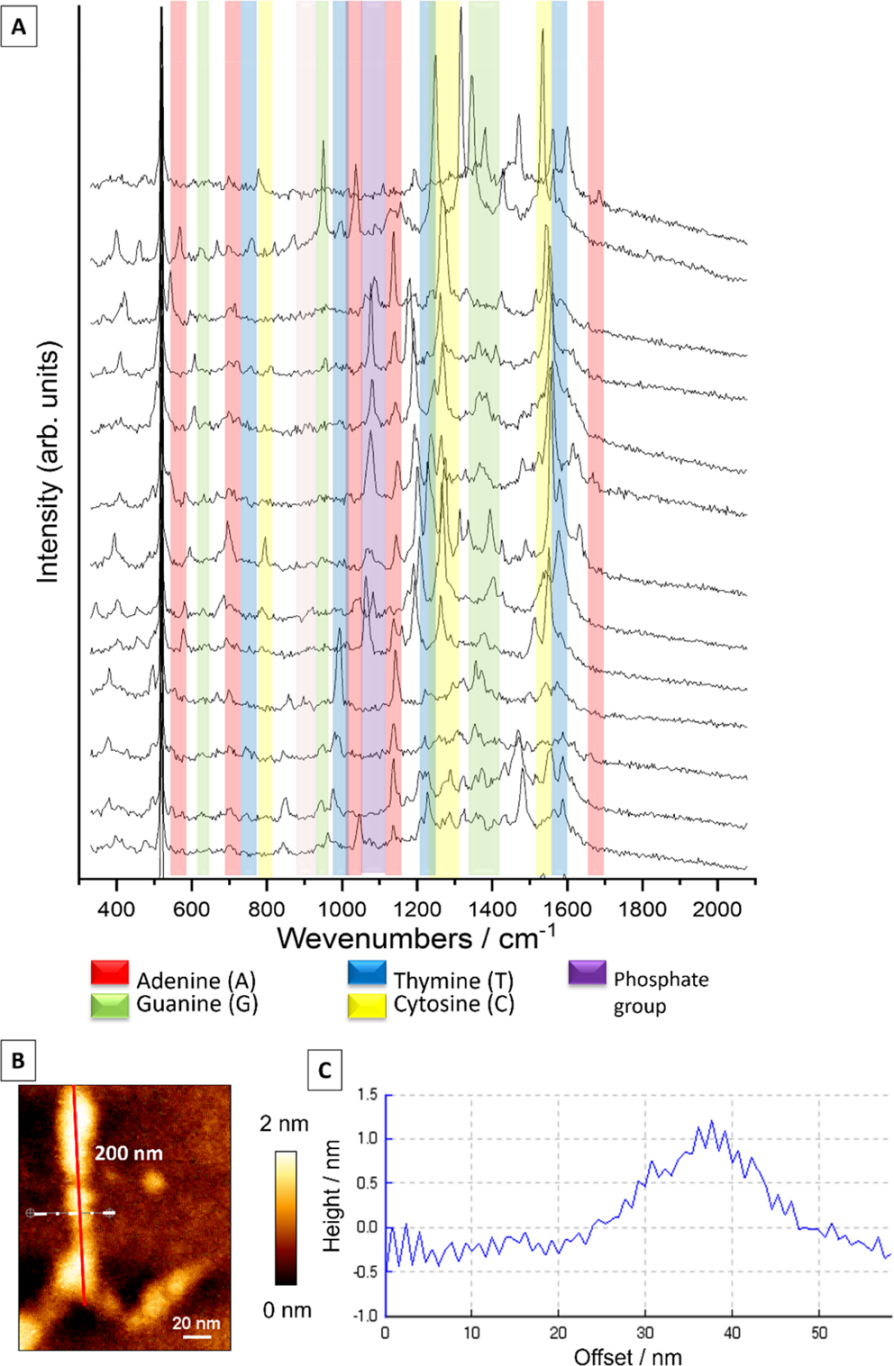
(A) TERS spectra of calf thymus DNA recorded along a 200 nm line along the strand (*t*_acq_ = 1 s; *P* = 700 μW at λ_exc_ = 532 nm) with marked characteristic Raman modes of the nucleobases. (B) The corresponding AFM topography was scanned during the TERS measurement (spectra were collected along the red line). (C) Profile across the DNA strand as indicated with a white dashed line in panel (B). The very intense peak at 520 cm^–1^ comes from the silicon signal.

In the TERS experiments, the sample was immobilized on positively charged mica (see Experimental Section), which should lead to a homogeneous orientation of the sample with outward pointing negatively charged phosphate moieties interacting with the substrate. In contrast to the previously discussed SERS experiment, in TERS, only molecules directly beneath the tip contribute to the signal, and a spatial resolution of a few nanometers and even beyond can be achieved.^8−10^ A differentiation of DOX-containing DNA regions from DOX-free regions should be feasible and correlate to height differences in the corresponding AFM topography images. In the first experiment, TERS spectra of untreated calf thymus DNA were acquired. Figure 4 shows selected/typical TERS spectra from the experiment. The characteristics of DNA can be detected in the spectra along the strand. Specifically, the different nucleobases can be identified by their ring breathing modes. This means that adenine (A), thymine (T), guanine (G), and cytosine (C) can be differentiated. This is demonstrated in the SERS spectra shown in Figure 3 and further reference data.^11,14,42^ TERS band position fluctuations of a few wavenumbers were observed and are a general phenomenon of a few molecule experiments.^59−63^

The spectra shown in Figure 4 were collected at equidistant measurement points along a 200 nm line with 0.25 nm of step size. Characteristic Raman modes of adenine (717–719 cm^–1^) and guanine (677 and 1315 cm^–1^) can be assigned. Bands at 872, 1026, and 1447 cm^–1^ indicate deoxyribose, and at 1094 cm^–1^, PO_2_. As indicated for high-resolution TERS experiments, spectra vary from point to point due to site-specific tip-molecule interactions, e.g., lower band intensities at 1023, 1091, and 1443 cm^–1^ (deoxyribose-related modes^48^) and higher intensity bands at 1313 and 1412 cm^–1^ (assigned to A^64,65^ or A/G and A/C, respectively^14,48^). A band at ca. 652 cm^–1^ (guanine ring breathing mode^36,42,47^), 872 cm^–1^ (deoxyribose), 957 (cytosine^65^), 1011 and 1061 cm^–1^ (deoxyribose^48^), 1284 cm^–1^ (cytosine^36,41,66^), 1711 cm^–1^ (guanine^48^) appears together with broad bands at 1342 and 1455 cm^–1^ (deoxyribose^48,66^), 1535, 1560, and 1607 cm^–1^ (cytosine^48,65^ or adenine^66^). Phosphate backbone-related bands were detected at 1090 cm^–1^ and at 1250 cm^–1.^^67^^67^ Deoxyribose-phosphate bands were located at ca. 850^51^ and 950–960.^48,51^Figure 4B shows the AFM topography image obtained during the TERS measurement. Figure 1S provides a scan with a higher resolution.

**Figure 5:**
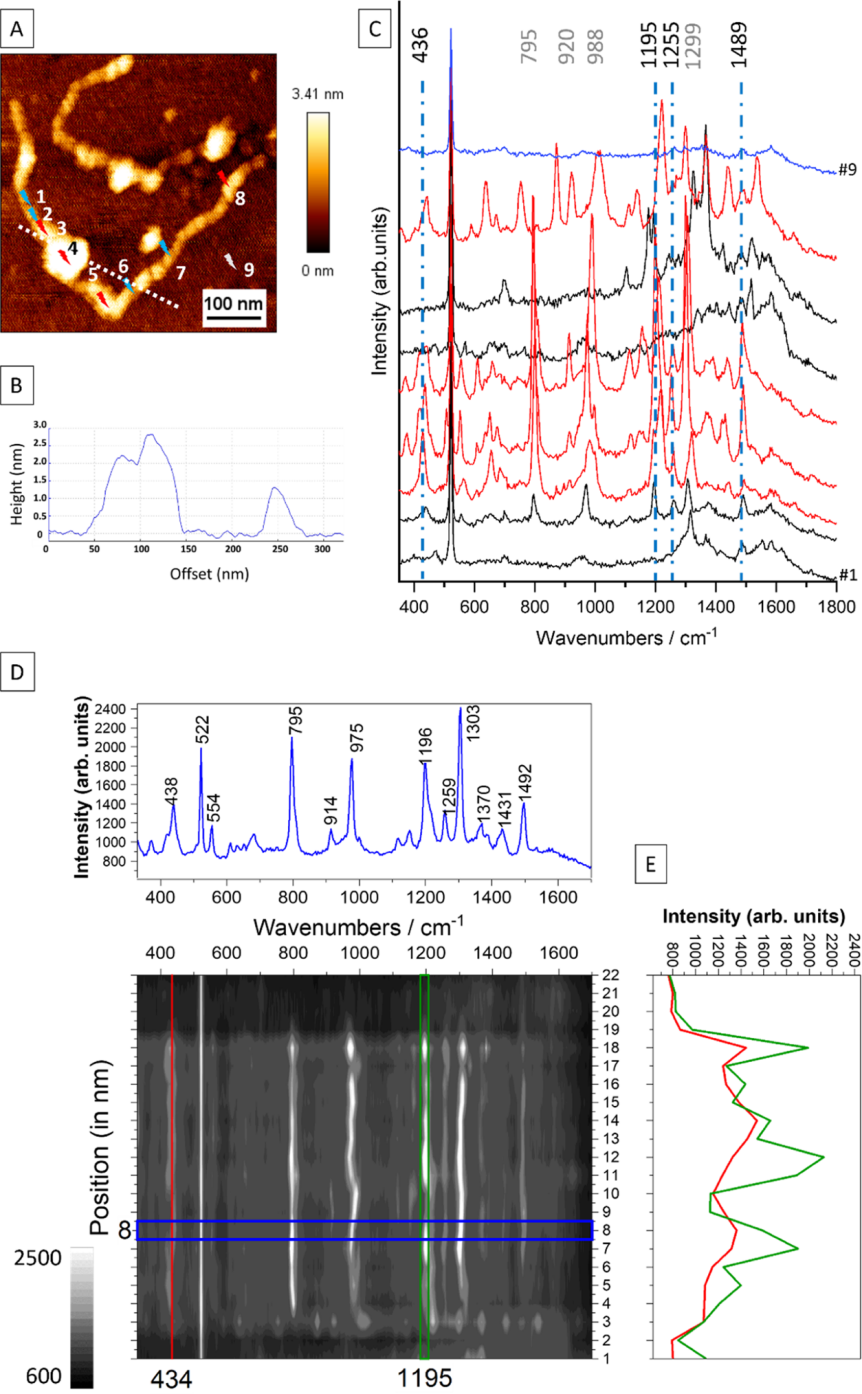
(A) AFM topography of DOX-treated double-stranded calf thymus DNA with a profile line given in panel (B) and with positions where TERS spectra were recorded. (C) Examples of TERS spectra recorded with *t*_acq_ = 1 s, *P* = 700 μW, λ = 532 nm. Arrows in panel (A) indicate spectra with (red) and without DOX (blue) signal. Raman bands marked by dotted blue lines are marker bands of DOX. The blue spectrum (#9) is collected on mica to exclude tip contamination. (D) TERS intensity map (position vs wavenumbers) recorded along the loop in panel (A) with contour plot profiles: TERS spectrum with DOX marker bands (blue). (E) Intensity progression of the dominating DOX marker bands at 434 cm^–1^ (red) and 1195 cm^–1^ (green) within the measured line.

As mentioned earlier, several SERS and Raman studies to interpret SERS and Raman spectra of DNA, as well as the DOX–DNA complex, have been published.^29,34,68^ Here, we present, for the very first time, highly localized TER spectra of a DNA–DOX complex correlating the identification of the drug and the location within a single DNA strand. The recorded TERS spectra show new bands that are not visible in the SERS spectra presented above, while other bands are absent. In Figures 5 and S5, TERS spectra recorded along a double-stranded calf thymus DNA–DOX complex are presented together with intensity progression of the dominating DOX marker bands at 434 and 1195 cm^–1^ along the DNA loops (Figure 5D,E). As was mentioned and reported in ref (69), the presence of DOX intercalation results in structural changes of the corresponding sites in the DNA strands, as shown in Figure 5A,B, such as formations of chain loops and overlaps. However, an increased height of the DNA strands cannot be understood as a simple sum of DNA strands and DOX height. If a single anthracycline molecule interacts with the DNA strands, the strand can relax, but if many molecules intercalate, the filaments appear more and more aggregated and entangled. In our experiments, we observe that DOX-containing spectra clearly correlate with the increased height of the DNA strand (Figure 5B). In other words, spectra recorded on DNA loops contain spectral markers of DOX, indicating incorporation of the drug in the DNA double strand. As DOX does not interact strongly with mica (in contrast to the silver substrate of the SERS experiment), it can be assumed that free DOX was not present and, consequently, was not detected. Figure 3 indicates that the SERS spectrum of free DOX differs from the DNA–DOX complex. The TERS spectra in Figure 5C show that the marker bands of DOX (to 1195, 1255, and 1489 cm^–1^) are shifted in comparison to unbound DOX SERS spectrum (1205, 1234, and 1439 cm^–1^) and were detected with lower intensity. This also applies to bands at 1235 cm^–1^ (1255 cm^–1^ in TERS) and 1435 cm^–1^, which can be assigned to aromatic ring stretching modes. The changes in DOX band intensities after complexation with the DNA, as seen in the TERS spectra, can be related to a selective short-range enhancement of certain vibrations situated closer to the surface of the TERS tip and oriented perpendicularly to it (according to the surface selection rule^69^). Since bands at 1235 cm^–1^ (1255 cm^–1^ in TERS) and 1435 cm^–1^ can be assigned to aromatic ring stretching modes, their lower intensities suggest that the chromophore was buried inside the DNA and therefore was no longer accessible for the TERS tip. At this time, not all of the spectra changes between SERS and TERS data can be explained in detail; however, it is clear that in the SERS case, the molecules will always try to minimize energy during adsorption to the SERS substrate, whereas in TERS, also thermodynamically unfavorable positions will be even more abundant if the specimen has no possibility to rearrange. The rearrangement is obviously hindered in our case. A further difference is the way of adsorption; clearly, there will be a difference between DNA adsorption to Ag or to mica. Since the DNA molecule was fixed to the mica surface, the D ring of DOX protruded from the DNA strand and interacted directly with the TERS tip, whereas the B and C rings were buried inside the DNA double helix. Their lower intensities suggest that the chromophore was buried inside the DNA and, therefore, was no longer accessible for the TERS tip.

In contrast, some bands (Figure 5C,D) were detected with increased intensity, i.e., at 976, 1303 (ν (C–O)^68^), and 1489 cm^–1^ (ring stretching^68^). Since the DNA molecule was fixed to the mica surface, the D ring of DOX protruded from the DNA strand and interacted directly with the TERS tip, whereas the B and C rings were buried inside the DNA double helix. Since the signal-enhancing ability of the metal apex in TERS is short-ranged (<5 nm), such a geometry should lead to a lower intensity of the bands associated with vibrations of the CCOCH and C=O exogenous groups (464, 1209, 1244, and 1449 cm^–1^), and in the spectra, the intensity clearly decreased in comparison with those of the skeletal modes.

The TER spectra of DOX–DNA also provided information about the DNA itself. The presence of DOX can be correlated with the detection of cytosine (795 and 1268 cm^–1^) and guanine (640–660, ∼1360 cm^–1^). The high band intensity of nucleobase modes further confirms the intercalation between guanine and cytosine DNA base pairs, which agrees with previously published data.

The results of this investigation can be divided into two major parts. First of all, we can localize the intercalation sites of DOX into calf thymus DNA on a single dsDNA level with nanometer precision based on the correlation of topographic and structural information provided by TERS experiments. Furthermore, the high lateral resolution directly confirms the preferred intercalation site of DOX, namely, between guanine and cytosine pairs. The structural changes evidenced by the SERS and TER spectra of the same complexes also point toward a substrate specificity of the DOX–DNA complexes after thermodynamically controlled adsorption to Ag (SERS) or mica (TERS), which is expected to affect the Raman features recorded from DNA or DOX–DNA complexes. As a more fundamental aspect, the spectral variations related to minute tip—sample position differences confirm recent theoretical and experimental evidence of the strong local dependence of chemical effects based on actual tip—sample location. This can be reflected in PCA results (Figure S4), which efficiently identify DOX intercalated into DNA and discern structural changes resulting from the intercalation process. However, the high spectral variability of TER spectra resulting from tip—sample position differences and molecule orientations makes this analysis inefficient. Based on the current results, the direct investigation of the actual intercalation of DOX into DNA with similar spectral and special resolution would be of great interest.

## Conclusions

For the first time, we present TER spectra from DNA complexed with DOX. The extremely high lateral resolution of the TER spectra allows to locate the intercalation sites of the DOX in the double-stranded calf thymus DNA. Indeed, the TERS results indicate a preferred intercalation of DOX molecules between the guanine and cytosine bases. Based on our results, the high-resolution TERS approach provides a novel experimental approach to investigate the site-specific molecular interaction of drugs with DNA. Consequently, it has great potential for qualitative and quantitative evaluation of the potential therapeutic effects of DNA-targeting drugs.

The TER spectra of DOX incorporated in the DNA strand show strong Raman features of the drug, particularly confirming DOX–DNA interaction. Moreover, the DOX signal correlates with C and G characteristics, which are specifically involved in the intercalation. The TERS signal related to the DOX–DNA complex is due to changes in DNA morphology/conformation, making nucleobases more accessible for the TERS tip. Spectra without DOX signals are consequently more complex and less intense, and the signal-to-noise ratio is lower.

In summary, we show that TERS can be successfully applied in a combined, highly localized, and structurally sensitive evaluation of drug–DNA interaction, correlating morphology and structure. It must be emphasized that the experiments were done under ambient conditions without further utilizing so-called gap modes. A transfer of this or similar systems even to a liquid environment is feasible, consequently providing the potential to perform dynamic investigations.

## Supporting Information Available

Detailed description of experimental procedures; representative AFM topography images of calf thymus DNA without (control DNA, A) and after incubation with 1 μM DOX (B) (Figure S1); time-dependent SERS measurements of DOX; spectra collected every 1s with 1 s acquisition time (700 μW excitation at λ_exc_ = 532 nm); linear background subtraction (Figure S2); TERS spectra recorded along a 40 nm line with a point-to-point step size of 1 nm (1 s acquisition time; with 700 μW excitation at λ_exc_ = 532 nm) with marked characteristic Raman modes of the nucleobases; selected spectra from this grid presented in Figure 4 (Figure S3) (PDF)
