# Supplementary material for: DOX-DNA Interactions on the Nanoscale: In Situ Studies Using Tip-Enhanced Raman Scattering
Source: Anal Chem. 2024 May 21;96(22):8905–13. doi: 10.1021/acs.analchem.3c05372 (PMC11154666; doi:10.1021/acs.analchem.3c05372)
Supplement: Supplementary file 1 — ac3c05372_si_001.pdf [file ac3c05372_si_001.pdf]

Supporting Information

DOX-DNA interactions on the nanoscale: in situ studies using tip-enhanced Raman scattering

Katarzyna Majzner[1], Tanja Deckert-Gaudig[2,3], Malgorzata Baranska[1,4], Volker Deckert[2,3]*

[1]Department of Chemical Physics, Faculty of Chemistry, Jagiellonian University, Gronostajowa 2, 30-387 Krakow, Poland;
[2]Friedrich Schiller University Jena, Institute of Physical Chemistry and Abbe Center of Photonics, Helmholtzweg 4, Jena 07743, Germany;
[3]Leibniz Insti-tute of Photonic Technology, Albert-Einstein-Str.9, Jena 07745, Germany;
[4]Jagiellonian Centre for Exper-imental Therapeutics (JCET), Jagiellonian University, Bobrzynskiego 14, 30-348 Krakow, Poland;
Email address of the corresponding author: volker.deckert@uni-jena.de

ABSTRACT: Chemotherapeutic anthracyclines, like doxorubicin (DOX), are drugs endowed with cytostatic activity and are widely used in anti-tumor therapy. Their molecular mechanism of action involves the formation of a stable anthracycline-DNA complex, which prevents cell division and results in cell death. It is known that elevated DOX concentrations induce DNA chain loops and overlaps. Here, for the first time, tip-enhanced Raman scattering (TERS) was used to identify and localize intercalated DOX in isolated double-stranded calf thymus DNA, and the correlated near-field spectroscopic and morphologic experiments clearly locate the DOX molecules in the DNA and provide further information regarding specific DOX-nucleobase interactions. Thus, the study provides a tool specifically for the identification of intercalation markers and in general for the analysis of drug-DNA interactions in general. The structure of such complexes down to the molecular level provides mechanistic information about cytotoxicity and the development of potential anticancer drugs.

Table of Contents

Experimental Procedures - S-2
Figure S1 - S-3
Figure S2 - S-4
Figure S3 - S-4
Figure S4 - S-5
Figure S5 - S-6
References - S-7
Author Contributions - S-8

Experimental Procedures

DNA preparation. Double-stranded and lyophilized fibrous DNA (Type XV) from calf thymus was dissolved in buffer, maintaining a physiological pH value, HEPES (20 mM, 4-(2-hydroxyethyl)-1-piperazineethanesulfonic acid) and magnesium chloride (20 mM, $MgCl_2$) to fix the strands with the phosphate moieties onto the mica (BAL-TEC) substrate. All chemicals were purchased from Sigma Aldrich, Germany. The DNA solution (c = 5 ng /µL) was heated to 40 °C for 10 min. 1-2 µL of the thermally treated DNA solution was then dropped onto a freshly cleaved mica sheet, incubated for 5 min, and dried in an argon atmosphere. Finally, samples were carefully washed twice with deionized water to remove the buffer and dried in an argon atmosphere. It is worth mentioning that the DNA sample was washed after deposition on mica to remove any free and unbounded DOX molecules from the solution.

AFM imaging. Standard AFM imaging was performed with a Nanowizard III instrument (JPK-Bruker) using intermittent-contact cantilevers (Tap190Al-G, Budget Sensors) with a scan rate of 0.8 Hz and a resolution of 256×256 pixels. Measurements were carried out under ambient conditions. For TERS imaging, standard non-contact AFM cantilever tips (NSG10, NT-MDT, r (tip) = 6–10 nm) were evaporated with 25 nm silver and stored under argon until use.

TERS measurements. TERS spectra were recorded along a double-stranded calf thymus DNA (dsDNA) molecule with a step size of 1 nm, if not mentioned otherwise. For illumination, a laser source with λ_{exc} = 532 nm (P = 750 µW, t_{acq} = 1 s) was used to excite Raman. The setup is based on an AFM (Nanowizard III, JPK, Germany) mounted onto an inverted microscope (Olympus IX 71, Japan). The light was focused and collected through a 60x oil immersion objective (N.A. 1.45). The general setup of the TERS instrument has been described in detail previously[1]. A schematic illustration of the experimental setup e is given in Fig. 1 A.

SERS measurements. For SERS experiments, DNA, DOX, and DNA/DOX samples were dissolved in 20mM HEPES (4-(2-hydroxyethyl)-1-piperazineethanesulfonic acid). 2-3 µL of each solution was applied to a silver island film and measured in liquid. The silver island film was prepared by evaporating 6 nm silver on glass coverslips (pre-cleaned with a HNO_3/H_2O_2 3:1 solution) and annealed at 300 °C for 60 s). The evaporation and procedure have been described in detail elsewhere.[2–4] SERS spectra were recorded at λ_{exc} = 532 nm (P = 750 µW, t_{acq} = 1-3 s).

.

.

Figure S1. Representative AFM topography images of calf thymus DNA without (control DNA, A) and after incubation with 1 µM DOX (B).

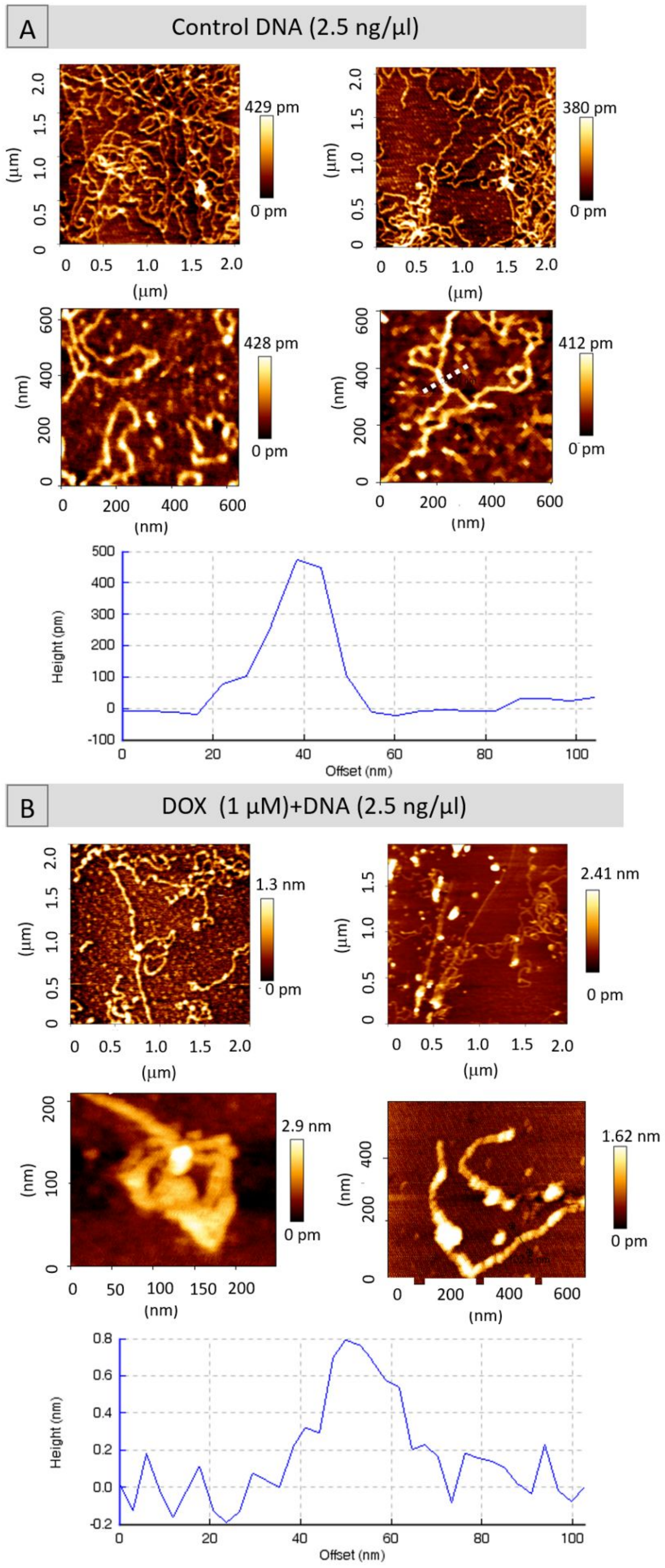

Figure S2. Time dependent SERS measurements of DOX. Spectra collected every 1s with 1 s acquisition time (700 μW excitation at λ_{exc} = 532 nm). Linear background subtraction was applied

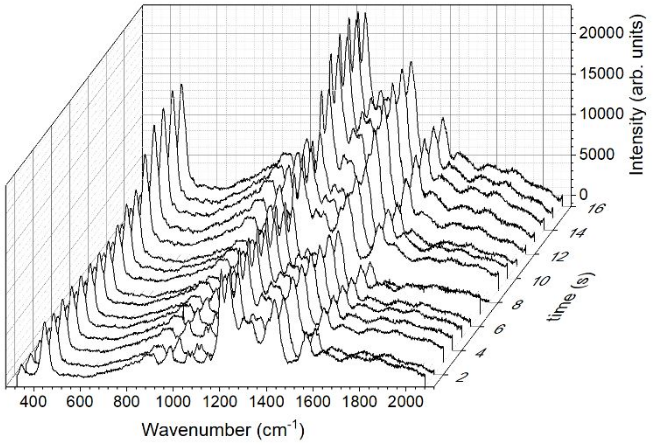

Figure S3. TERS spectra recorded along a 40 nm line with a point-to-point step-size of 1 nm (1 s acquisition time; with 700 μW excitation at λ_{exc} = 532 nm) with marked characteristic Raman modes of the nucleobases. Selected spectra from this grid are presented in Fig. 4.

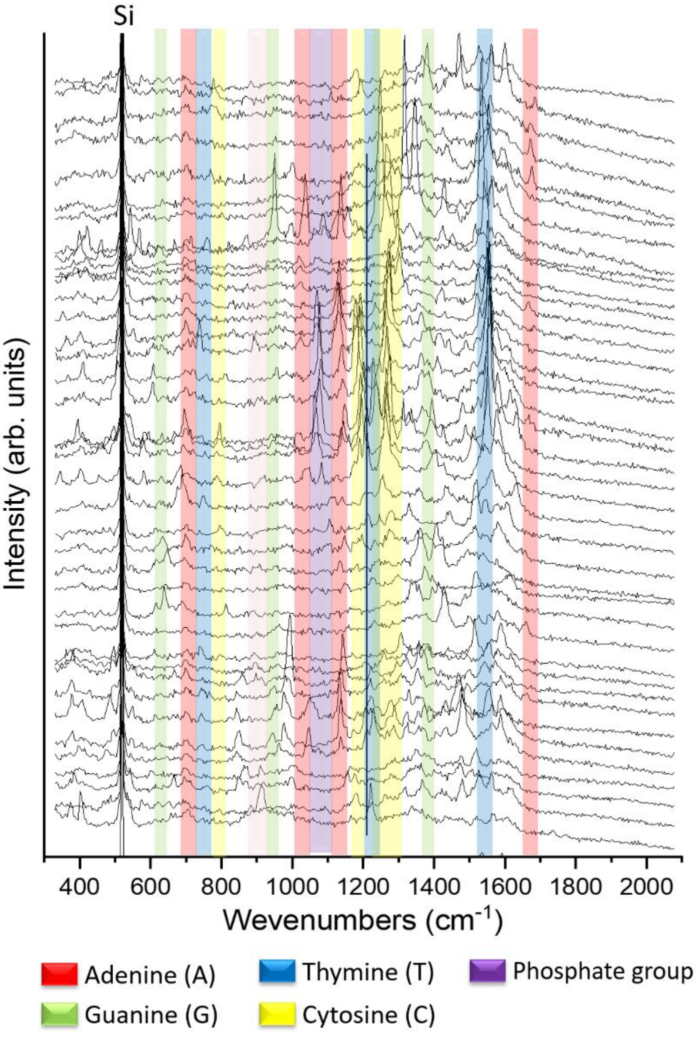

Figure S4. PCA on TERS spectra from DNA and DOX-DNA. (A and B) Scores and (C) loadings of the first two principal components (PC) resulting from the PCA of the vector-normalized TER spectra over the range 350-500 and 530-1750 cm^{-1}. The first two principal components (PCs) and PC-4of PCA shows the clustering of the TER spectra of DNA (99 spectra) and DOX-DNA samples (500 spectra). (B) The corresponding loading plots of PC 1 and PC 2. Points that are distant from the main clusters (along PC-1) exhibit strong DOX signal intensities. DOX marker band at 436, 1200 cm^{-1} are observed on loadings together with cytosine (793 cm^{-1}).

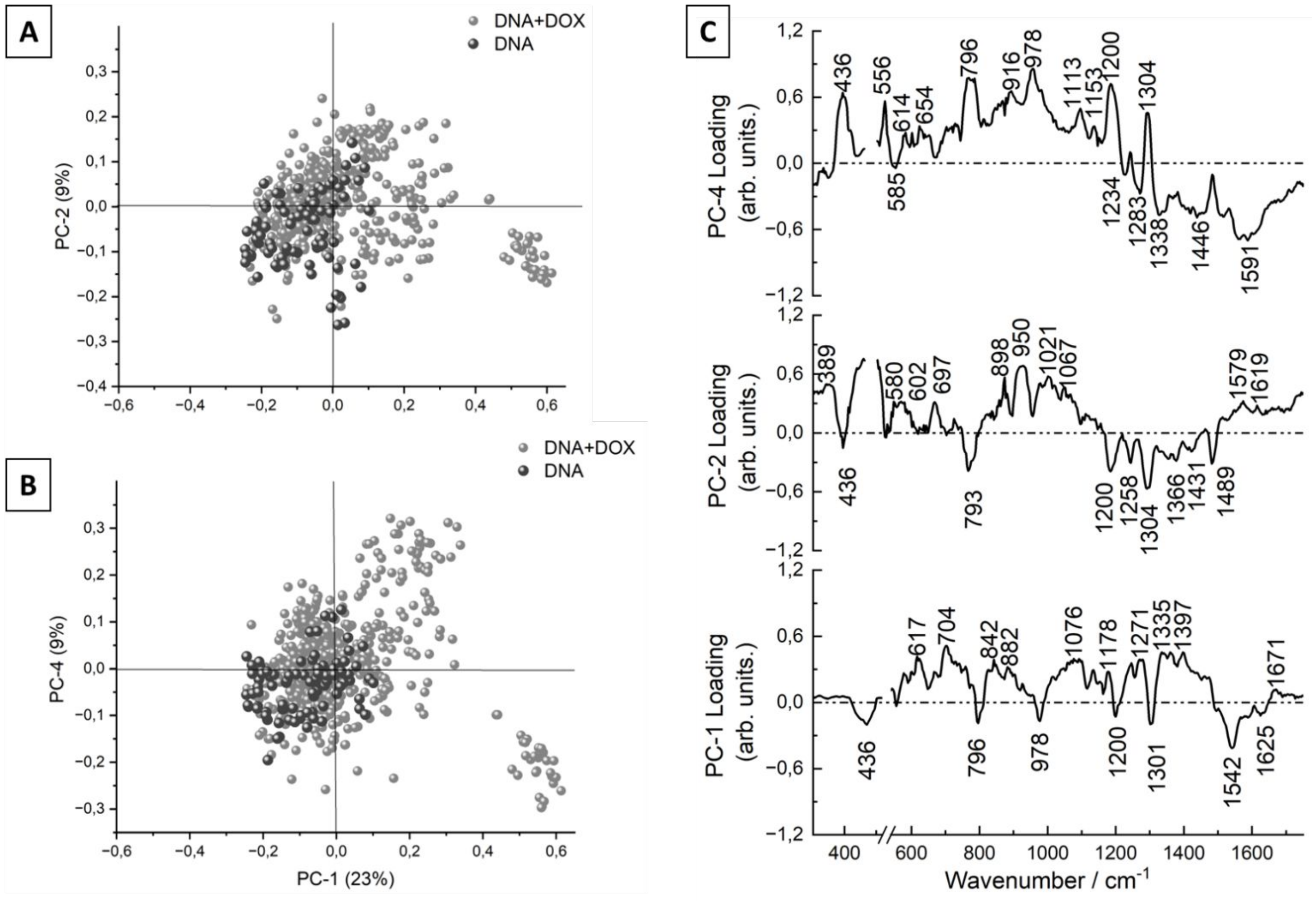

Figure S5. TERS spectra recorded with a point-to-point step-size of 1 nm (1 s acquisition time; with 700 μW excitation at λ_{exc} = 532 nm) with marked characteristic Raman modes of the DOX.

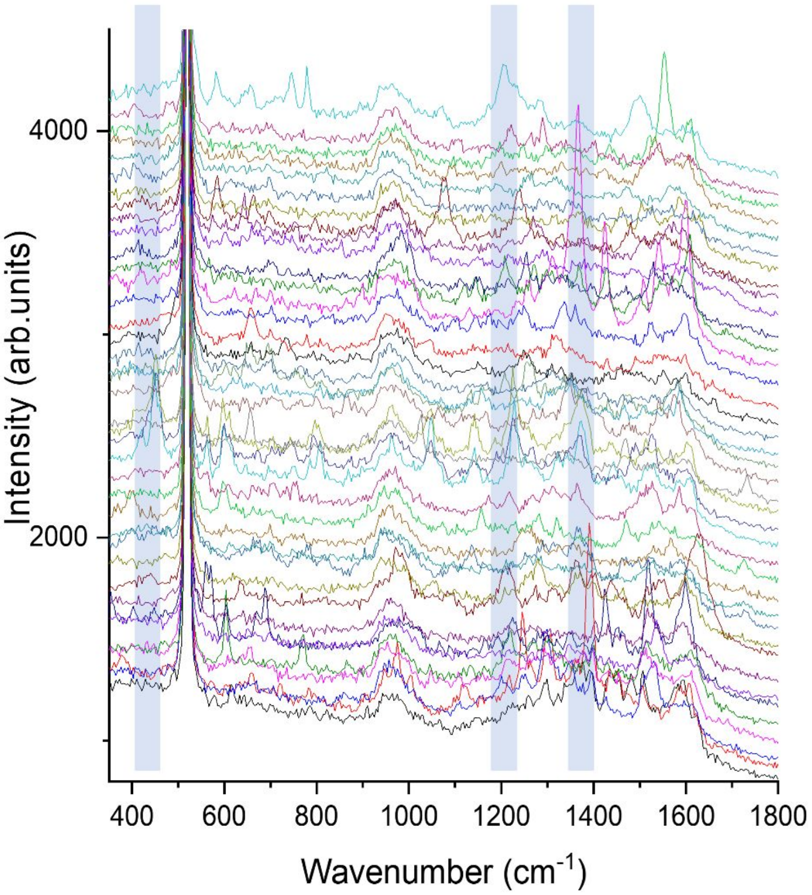

References

(1) Langelüddecke, L.; Singh, P.; Deckert, V. Exploring the Nanoscale: Fifteen Years of Tip-Enhanced Raman Spectroscopy. *Applied Spectroscopy* **2015**, *69* (12), 1357–1371.

(2) Feofanov, A.; Ianoul, A.; Kryukov, E.; Maskevich, S.; Vasiliuk, G.; Kivach, L.; Nabiev, I. Nondisturbing and Stable SERS-Active Substrates with Increased Contribution of Long-Range Component of Raman Enhancement Created by High-Temperature Annealing of Thick Metal Films. *Analytical Chemistry* **1997**, *69* (18), 3731–3740.

(3) Deckert, V.; Fokas, C.; Zenobi, R. Controlled Formation of Isolated Silver Islands for Surface-Enhanced Raman Scattering. **2000**, *54* (11), 1577–1583.

(4) Rasmussen, A.; Deckert, V. Surface- and Tip-Enhanced Raman Scattering of DNA Components. *Journal of Raman Spectroscopy* **2006**, *37* (1–3), 311–317.

Author Contributions

KM, VD, and MB conceived and designed the research; KM performed sample preparations and SERS measurements; KM and TDG performed TERS measurements and analyzed the data. KM prepared the figures and prepared the original and revised manuscript. The manuscript was written through the contributions of all authors. All authors have given approval to the final version of the manuscript.
